# Comparative analysis of the RVA VP7 and VP4 antigenic epitopes circulating in Iran and the Rotarix and RotaTeq vaccines

**DOI:** 10.1016/j.heliyon.2024.e33887

**Published:** 2024-07-04

**Authors:** Tina Fallah, Roxana Mansour Ghanaiee, Abdollah Karimi, Seyed Mohsen Zahraei, Sussan Mahmoudi, Masoud Alebouyeh

**Affiliations:** a Department of Microbiology, Faculty of Biological Sciences, Alzahra University, Tehran, Iran; b Pediatric Infections Research Center, Research Institute for Children's Health, Shahid Beheshti University of Medical Sciences, Tehran, Iran; c Center for Communicable Diseases Control, Ministry of Health and Medical Education, Tehran, Iran

**Keywords:** Rotavirus, Vaccine, *VP4*, *VP7*, Lineage, Antigenic epitopes

## Abstract

Analyzing the lineages and detecting antigenic variation in immunogenic motifs of Group A Rotavirus (RVA) variants is crucial because it can impact vaccine efficacy. This study investigated the circulating lineages of VP4 and VP7 proteins of human RVA isolates and their phylogeny in ≤24-month-old symptomatic, rotavirus-positive children with transudative diarrhea within 48 h of admission to Mofid Children's Hospital between December 2020 and March 2022 in Tehran, Iran. Antigen detection was performed by ELISA, RNA extraction, and semi-nested multiplex PCR for G/P genotypes, followed by sequencing and bioinformatic analysis using multiple sequence alignments in MEGA and phylogenetic analysis by Geneious Prime. The similarity of VP7 and VP4 amino acids with the RotaTeq and Rotarix vaccine strains for cytotoxic T cell and antigenic epitopes was evaluated using the UCSF Chimera Molecular Modeling System. Overall, 27.3 % of the samples were RVA positive, showing untypeable (2.5 %), single (76.9 %), and mixed (20.5 %) genotypic characteristics. The strains clustered in the G1/II, G2/IV, G3/I, G4/I, G9/III, P (Kachooei et al., 2023) [8]/III, P (Howley et al., 2020) [4]/V, and P (Wahyuni et al., 2021) [6]/I lineages. Comparative analysis of VP7 antigenic epitopes showed that the G1/II strains were completely conserved, while the G2/IV, G3/I, G4/I, G6, G9/III strains contained 2, 3–5, 2, 4 and 9 amino acid substitutions, respectively. The P (Kachooei et al., 2023) [8]/III genotypes differed by 3 amino acids, while the P (Wahyuni et al., 2021) [6]/I genotype had the most substitutions. CTL epitopes were completely conserved in G3/I strains, but other genotypes differed by 1–4 amino acids compared to the vaccine strains. Given the diversity of circulating RVA genotypes and the observed mutations in neutralizing and CTL epitopes, immune escape by some of the strains is likely in Iran. This finding underscores the importance of evaluating the effect of rotavirus vaccines on local genotypes and related lineages before implementing a vaccination program.

## Highlights

• The RVA G6 genotype was characterized in this study for the first time in Iran.
• Detected lineages were G1/II, G2/IV, G3/I, G4/I, G9/III, P [6]/III, P [4]/V, P [7]/I.
• G9 strains showed the highest amino acid substitutions in all 3 antigenic epitopes.
• CTL epitopes were conserved in G3 strains, but other genotypes differed by 1–4 aa.

## 1 Introduction

Rotavirus is a leading cause of diarrheal disease, accounting for approximately 29.3 % of global deaths related to diarrhea in children under 5 years of age [1]. Over 90 % of these mortalities occur in low- and middle-income countries. Although the global burden of rotavirus has significantly decreased over the past three decades due to the introduction of rotavirus vaccines, its prevalence remains high in Africa, Oceania, South Asia, and the Middle-East [2,3].

The most common rotavirus genotypes found in children worldwide vary by region and time period. This diversity is linked to the virus's genomic structure and geographic evolution, influenced by common host factors and transmission routes. The infectious RVA particle has an icosahedral structure with three layers and comprises 11 segments of double-stranded RNA that encode six structural proteins (VP) and six non-structural proteins (NSP) [4]. To date, 42 G genotypes and 58 P genotypes have been described for group A rotaviruses [5]. The most prevalent genotypes worldwide are G1P [6], G2P [4], G3P [6], G4P [6], G9P [6], and G12P [7,6]. These genotypes are determined based on the two outer capsid proteins, VP7 (G type) and VP4 (P type), which function as specific binding sites for neutralizing antibodies [4]. According to recent studies, G1P [6], G9P [4], and G9P [6] are the most prevalent circulating genotypes in Iran [8,6]. The introduction of rotavirus vaccines has been associated with changes in the distribution of rotavirus genotypes in certain areas, resulting in a decline in the prevalence of vaccine-targeted genotypes and an increase in the prevalence of genotypes not covered by the vaccines [9].

Recent studies have revealed variations in the distribution of RVA genotypes across different age groups. In Canada and Greece, a higher prevalence of G12P [6] and G9P [6] genotypes was observed among older children, specifically those aged between 24 and 59 months [10,11]. In children aged 0–12 months, genotypes such as G1P [6] and G9P [4] were found to be more common [12,13]. Several factors may contribute to these variations, including differences in the expression of certain receptors in the intestinal tract at different age groups, exposure to common genotypes in different geographic areas, induced immunity, and host genetics [[14], [15], [16]]. Similarly, there is evidence of a correlation between rotavirus genotypes and disease symptoms. Studies have shown that different genotypes of rotavirus are associated with varying clinical characteristics and disease severity. In one study, G1P [6] and G9P [6] were the most common genotypes detected in children with moderate and severe acute gastroenteritis (AGE), respectively [12]. A significantly higher frequency of fever was reported in children infected with the G3P [6] genotype, according to Mathew et al. [17]. This correlation was also detected in the case of emerging genotypes, such as G8P [6] and G9P [4,18].

The interaction between RVA antigens of various genotypes and the host immune system offers an explanation for variations in disease outcomes among immunized and non-immunized children. This interaction primarily depends on the immunogenic domains within the VP4 and VP7 proteins. Notably, the VP7 gene, which encompasses 326 amino acids, contains nine variable regions, including four antigenic epitopes identified as 7-1a, 7-1 b, and 7–2. Activation of the VP4 protein, spanning 776 amino acids, requires proteolytic cleavage into two distinct segments: VP8 and VP5. Each segment contains its own set of antigenic epitopes, with VP8 having four (8-1–8-4) and VP5 having five (5-1–5-5) [4]. Due to the diversity of immunogenic motifs within these proteins, multiple lineages and sub-lineages have been defined. According to Motamedi-Rad et al., among the predominant G-types, there are eleven, six, four, six, and six lineages for G1, G2, G3, G4, and G9 lineage, respectively. In terms of P serotypes, four, five, and five distinct lineages were reported for P [6], P [4], and P [7], respectively [19].

RV vaccination is the most effective strategy for significantly reducing the incidence of severe infections and the number of deaths in children. Rotarix (GlaxoSmithKline) consists of a monovalent G1P [6] strain derived from a single human G1P [6] strain. Conversely, RotaTeq (Merck) is a human-bovine reassortant vaccine, composed of five strains derived from human (G1, G2, G3, and G4) and bovine (P [6]) strains [20]. Both RVA vaccines have demonstrated the ability to elicit homotypic and heterotypic immune responses, resulting in a notable decline in morbidity and mortality. However, the RVA vaccine has proven to be more effective in high-income countries (80%–90 %) against severe rotavirus disease compared to low- and middle-income countries (40%–70 %) [21]. Various factors contribute to this disparity, including the genotypic and lineage diversity of RVA strains, which are driven by recombination, rearrangement, reassortment, and mutations. Additionally, host genetic factors, malnutrition, gut microbiota dysbiosis, co-infections, environmental enteropathy, and the passive transfer of maternal antibodies have been suggested as potential determinants of differences in RVA vaccine efficacy between high- and low-income countries [19]. RVA lineages with distinct antigenic properties could potentially allow RVA strains to evade vaccine-induced immunity [22].

With the increasing diversity of RVA strains and the incidence of uncommon fully and partially heterotypic genotypes, such as G1P [7] or G9P [4], particularly in countries without RVA vaccination programs, concerns are growing regarding the reduced effectiveness of approved vaccines against the emerging variants [21]. Iran is among the countries that have not yet introduced the RVA vaccine, and there is lack of data about circulating lineages of RVA genotypes and their compatibility with the common vaccines' strains. To choose the most appropriate vaccine that would provide the highest coverage, ongoing research is being conducted nationwide to determine the percentage of children infected with RVA, identify the predominant circulating genotypes, and assess the prevalent lineages. In this cross-sectional study, the circulating lineages of VP7 and VP4 proteins of human rotavirus A (RVA) strains were analyzed in ≤24-month-old symptomatic children with transudative diarrhea within 48 h of admission to Mofid Children's Hospital in Tehran, Iran. Additionally, the amino acid sequences of these RVAs and their potential antigenic distinctions and cytotoxic T cell epitopes compared to the vaccine strains were assessed to monitor the mutations in the RVA strains and to choose the best policy for RVA vaccination in Iran.

## 2 Materials and methods

### 2.1 Sample selection

In order to determine the phylogenetic and antigenic epitope associations of VP7 and VP4among the Iranian and vaccine strains, 51 out of 187 stool samples from patients with transudative diarrhea with a positive stool antigen test for RVA were included in this study. The stool antigen test was conducted using the ProSpecT™ Rotavirus kit, (Oxoid, UK) as described previously [8].The samples were obtained within 48 h of admission from symptomatic children aged ≤24 months at Mofid Children's Hospital in Tehran, Iran, between December 2020 and March 2022. This study was approved by the research ethics committees of the Research Institute of Children's Health at Shahid Beheshti University of Medical Sciences (IR.SBMU.RICH.REC.1401.021), and parental consent was obtained for all the children's samples.

### 2.2 RNA extraction, RT-PCR, and amplification of VP7 and VP4

Viral RNA was extracted from 10 % stool suspensions of selected rotavirus-positive fecal specimens using the High Pure Viral Nucleic Acid Extraction kit (Roche, Mannheim, Germany). The RNA extracts were denatured at 95 °C for 5 min. Then, the AddScript cDNA Synthesis kit (Addbio, Daejeon, South Korea) was used to generate cDNAs using primers Beg9 and End 9 (VP7), and Con3 and Con 2 (VP4), following World Health Organization protocols No. 12 and 13 [23]. Next, semi-nested multiplex polymerase chain reaction (PCR) genotyping was conducted using specific primers as previously described [8]. PCR products were verified using electrophoresis in a 1.5 % agarose gel, and genotypes were identified according to the size of the PCR products, in line with WHO standard protocols.

### 2.3 Nucleic acid sequencing

After genotyping, PCR products from samples with single genotype infections that showed high-quality, single bands for VP7 and VP4 were considered suitable for sequencing. These PCR products were sent to a qualified company for sequencing of full-length VP7 and partial-length VP4 using the same forward primers from the first-round PCR (Beg9 and Con3 for VP7 and VP4, respectively) on the ABI3730xl sequencer with AB sequencing reagents. Quality control of the samples was conducted according to the company's quality value standards. For samples with mixed genotypes, only the single VP7 or VP4 segments were sequenced. Samples with two distinct G and P types were not sequenced (Table 1). The obtained sequences were analyzed using Chromas 2.2.6, and the sequences were manually checked and compared to other sequences in Genbank. Subsequently, their genotypes were confirmed using the web-based Rotavirus A genotyping tool (https://www.rivm.nl/mpf/typingtool/rotavirusa/). The nucleotide sequences were then submitted to GenBank, where they were assigned accession numbers OQ789844-OQ789866 for VP7 and OQ789867-OQ789894 for VP4.

**Table 1:** The characterized RVA genotypes using semi-nested multiplex-PCR and sequencing techniques in Tehran.

Semi-nested multiplex-PCR genotyping	Number^a^	No. sequenced^b^	Confirmed genotypes^c^	Changed genotypes^d^
G3 P [4]	2	2	2/2	None
G1 P [7]	5	5	2/2 G/[P]^e^, 3/3 [P]^f^	None
G1 P [6]	8	8	7/8 G/[P], 1/1 G	1/8, G6P [6]
G1G3 P [7]	3	3	3/3 [P]	None
G1G3 P [6]	1	1	1/1 [P]	None
G1G3 P [6]P [7]	1	0	not sequenced	None
G1G9 P [4]	1	1	1/1 [P]	None
G2 P [4]	1	1	1/1 G/[P]	None
G3 P [6]	4	4	1/1 G/[P], 2/2 G, 1/1 [P]	None
G3G9 P [6]P [7]	1	0	not sequenced	None
G4 P [6]	2	2	2/2 G/[P]	None
G8G9 P [6]	1	0	not sequenced	None
G9 P [4]	2	2	1/1 G^f^, 1/1 [P]	None
G9 P [6]	2	2	1/1 G, 1/1 [P]	None
G9 P [7]	4	4	1/1 G/[P], 2/2 G, 1/1 [P]	None
Gx P [x]	1	0	not sequenced	None

a The number of genotypes identified by PCR according to the WHO protocol.

b The number of RVA strains that were sent for VP7 and VP4 sequencing using the Beg9 and Con3 primers for VP7 and VP4, respectively. For strains with mixed genotypes, only the VP7 or VP4 segments with a single genotype (G or P) was sequenced. Sequencing was not done for samples with two distinct G and P types (GxGxP [x]P [x]) or those with low-quality PCR products.

c The number of RVA strains that showed the same G and P PCR-types (N) after sequencing (n) (n/N).

d The number of changes in genotypes after sequencing.

e G/[P]: The sequencing results revealed the same G and P types as the PCR results.

f [P], G: The sequencing only confirmed the G or P type, as the other segment (VP7 or VP4) had a low-quality PCR product.

### 2.4 Statistical analysis

The association of genotypes with clinical symptoms was analyzed using the Mann-Whitney test in GraphPad version 9.5.0, and a *p-*value of <0.05 was considered significant.

### 2.5 Phylogenetic analysis

For the phylogenetic analysis, nucleotide sequences of the VP7 and VP4 genes of related strains, as well as Rotarix and RotaTeq vaccine strains, were obtained from GenBank. Multiple sequence alignments were done using CLUSTALW in MEGA software Version X. Phylogenetic trees were constructed using the Maximum Likelihood method with the RAxML plugin version 8.2.11 in Geneious Prime software version 20, with the following parameters: Nucleotide model: GTR GAMMA, Algorithm: Rapid Bootstrapping and search for the best-scoring ML tree, Parsimony random seed:1. The results were validated using the bootstrap method with 1000 replicates. VP7 and VP4 amino acid sequence similarities with vaccine sequences were calculated using ClustalW in the Clustal Omega online tool [24].

### 2.6 Analysis of the VP7 and VP4 antigenic epitopes

To further investigate the discrepancies found in the antigenic epitopes of the circulating rotavirus strains in Iran, the amino acid changes within the antigenic epitopes of the RVA outer-capsid proteins VP7 and VP4 in Iranian strains were compared with the Rotarix and RotaTeq vaccine strains. Structural analysis of VP7 (PDB 3FMG) and VP8 (PDB 1KQR) was performed using the UCSF Chimera Molecular Modeling System software version 1.17.1.

## 3 Results

### 3.1 Patients and samples

In this study, 51 stool samples from patients admitted to Mofid Children's Hospital were primarily approved for genotyping and lineage determination based on their quality and quantity. These samples were from boys (37/51, 72.5 %) and girls (14/51, 27.4 %), aged ≤24 months (mean age = 14 months). The samples were collected across different seasons as follows: winter (25/51, 49 %), autumn (12/51, 23.5 %), spring (11/51, 21.5 %), and summer (3/51, 5.9 %) [8]. All the patients showed signs and symptoms of RVA infection at the time of admission to the emergency room at Mofid Children's Hospital [8].

### 3.2 Diversity of RVA genotypes and their association with clinical symptoms

Thirty-nine out of the 51 selected samples, excluding those that did not provide the first-round PCR product and second-round genotype-specific products, were genotyped. Details about the genotypes characterized by the conventional method are presented in Table 1. No significant association was found between clinical symptoms—fever, vomiting, dehydration, the severity of diarrhea—and defined genotypes (G1P [6], G1P [7], and uncommon genotypes) (Table 3). All children infected with the genotypes G3P [4] (n = 2), G9P [6] (n = 2), G9P [7] (n = 4), G6P [6] (n = 1), G1P [7] (n = 5), and G4P [6] (n = 2) experienced fever. However, 25 % (n = 1/4) of children with G3P [6] and 28.6 % (n = 2/7) of children with G1P [6] did not have fever. Children infected with G3P [4] did not experience nausea, while all children with G9P [7], G9P [6], G4P [6], and G1P [7] infections exhibited higher instances of nausea. Of the children infected with G1P [6], only 14.3 % (n = 1/7) showed signs of vomiting. Aside from 25 % (n = 1/4) and 20 % (n = 1/5) of children infected with G3P [6] and G1P [7], respectively, all other children infected with other genotypes were dehydrated. Only 25 % (n = 1/4) of children infected with the G3P [6] genotype had severe diarrhea (the stool form was watery), whereas other single- and mixed-genotype infections resulted in mild diarrhea (the stool form was semi-solid). None of the G2P [4] infections were associated with fever. Among patients with mixed infections, 40 % (n = 2/5) did not experience fever or nausea, yet all were dehydrated.

**Table 2:** Amino acid sequence identity matrix for comparing G and P types of Iranian strains with vaccine strains.

G and P genotype/lineage	G1/II Rotarix	GI/III RotaTeq	G2/II RotaTeq	G3/II RotaTeq	G4/I RotaTeq	G6 RotaTeq	P [6]/I Rotarix	P [6]/II RotaTeq
G1/II (n = 9)	96.39–96.92	93.77–94.30	74.43–75.69	79.02–80.31	76.10–76.90	79.61–80.31		
G2/IV (n = 1)	73.95	74.60	94.86	73.95	70.74	75.24		
G3/I (n = 5)	80.57–81.60	81.21–81.90	75.08–76.07	96.39–97.24	74.52–75.41	82.80–83.44		
G4/I (n = 2)	76.99	76.07	72.70	74.85	96.32	78.22		
G6 (n = 1)	82.67	82.00	76.67	85.67	79.00	93.00		
G9/III (n = 5)	80.19–81.65	79.55–80.98	77.92–79.14	85.25–86.15	78.53–79.75	83.12–83.86		
P [6]/III (n = 13)							92.34–93.09	94.35–94.95
P [4]/V (n = 5)							85.61–85.92	87.27–87.41
P [7]/I (n = 10)							70.07–71.73	68.00–69.61

**Table 3:** Association between clinical symptoms and RVA genotypes in infected children.

**Symptoms**	Genotypes			*p*-value^b^
	G1P [6] (n = 7)	G1P [7] (n = 5)	Uncommon genotypes^a^ (n = 23)	
**Fever**	5, 71.4 %	5, 100 %	18, 78.2 %	0.3
**Vomiting**	6, 85.7 %	5, 100 %	16, 69.5 %	0.4
**Dehydration**	7, 100 %	4, 80 %	22, 95.6 %	0.1
**Semi-solid Diarrhea**	7, 100 %	5, 100 %	22, 95.6 %	0.1

a Due to the small number of samples, uncommon genotypes with fewer than 5 observations, were considered as one variable. G3P [4] (n = 2), G3P [6] (n = 4), G6P [6] (n = 1), G2P [4] (n = 1), G4P [6] (n = 2), G9P [4] (n = 2), G9P [6] (n = 2), G9P [7] (n = 4), G1G3P [7] (n = 3), G1G3P [6] (n = 1), G1G9P [4] (n = 1).

b The Non-parametric T-test (Mann-Whitney) was used for this analysis.

### 3.3 Phylogenetic analysis and comparison of VP7 proteins of circulating Iranian RVA strains and vaccine strains

**Fig. 1:**
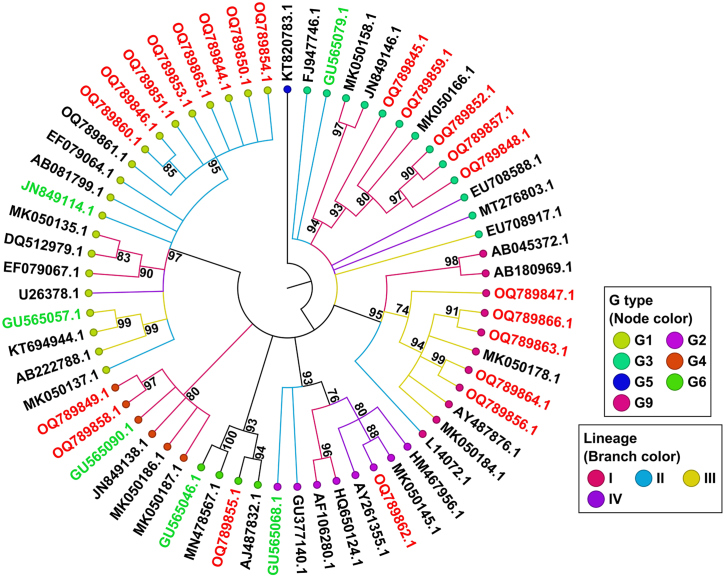
Phylogenetic analysis of the VP7 proteins of circulating Iranian strains and vaccine strains (Rotarix G1, RotaTeq G1-G4, and G6). Maximum Likelihood tree was constructed with the RAxML plugin in Geneious Prime, with the following parameters: Nucleotide model: GTR GAMMA, Algorithm: Rapid Bootstrapping and search for the best-scoring ML tree, Parsimony random seed: 1. KT820783.1 porcine strain was used as an outgroup. Vaccine strains and the study strains are shown in green and red, respectively. Bootstrap values (1000 replicates) of over 70 % are indicated.

Out of 39 samples with defined genotypes (30 single infections, 8 mixed infections, and one untypeable (GxP [x])), PCR products of 35 samples were approved for sequencing and lineage determination. As described in Table 1, the excluded samples did not show high-quality PCR products or presented co-infection with two distinct G and P types. A VP7 phylogenetic tree depicting the circulating Iranian strains, along with those of RotaTeq, Rotarix, and reference strains, is presented in Fig. 1. The analysis revealed that the Iranian RVA sequences corresponded to G-types G1 (n = 9), G2 (n = 1), G3 (n = 5), G4 (n = 2), G6 (n = 1) and G9 (n = 5), which were confirmed through similarity with reference isolates. G6 was characterized in this study for the first time in Iran.

The phylogenetic analysis indicated that G1 strains clustered in lineage II (Fig. 1). These strains exhibited amino acid sequence identities of 96.39 %–96.92 % compared with Rotarix G1 (lineage II) and 93.77 %–94.30 % in comparison to RotaTeq G1 (lineage III) (Table 2). The G2 strain belonged to lineage IV (Fig. 1), which was relatively distant from Rotarix G1/II, but similar to RotaTeq G2/II, with 73.95 % and 94.86 % similarity, respectively (Table 2). While the Iranian G3 strains were in different lineages compared to the vaccine strains (lineage II as opposed to lineage I), they showed high similarity, with 80.57 %–81.60 % and 96.39 %–97.24 % similarity to the G1/II strain of Rotarix and G3 lineage II strain of RotaTeq, respectively (Table 2). The G6 strain was relatively closely related to G1/II and G6 of Rotarix and RotaTeq strains, with 82.67 % and 93 % amino acid identities, respectively. The G4/I Iranian strains clustered in the same lineage as the RotaTeq G4 vaccine strain and demonstrated higher amino acid homology (96.32 %) compared to the G1/II of Rotarix (76.99 %). Additionally, G9 strains were included in lineage III (Fig. 1), and showed 80.19 %–81.65 % and 79.55 %–86.15 % identity with the G1 of Rotarix and G1-G4 and G6 of RotaTeq (due to the absence of G9 in both vaccines), respectively. Notably, the G3/I strains exhibited the highest amino acid identity with the G3/II of RotaTeq (96.39 %–97.24 %) (Table 2).

### 3.4 Phylogenetic analysis and comparison of VP4 (VP8) proteins of circulating Iranian RVA strains and vaccine strains

**Fig. 2:**
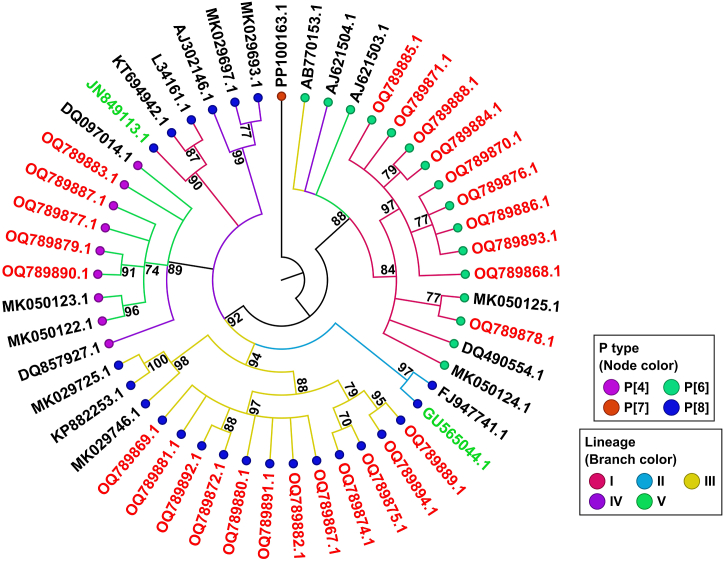
Phylogenetic analysis of the VP4 proteins of circulating Iranian strains and vaccine strains (P [6] strains of Rotarix and RotaTeq). Maximum Likelihood tree was constructed with the RAxML plugin in Geneious Prime, with the following parameters: Nucleotide model: GTR GAMMA, Algorithm: Rapid Bootstrapping and search for the best-scoring ML tree, Parsimony random seed: 1. PP100163.1 porcine strain was used as an outgroup. Vaccine strains and the study strains are shown in green and red, respectively. Bootstrap values (1000 replicates) of over 70 % are indicated.

A Maximum Likelihood tree was generated using partial genome sequences from the VP4 gene segment. The total number of nucleotide sequences acquired for P-types were 13, 5, and 10 for P [6], P [4], and P [7], respectively. The constructed phylogenetic tree revealed that Iranian P [6] strains fell into lineage III (Fig. 2), in contrast to Rotarix and RotaTeq P [6] strains, which are related to lineages I and II, respectively. These strains showed a higher similarity to the P [6] strain of RotaTeq, with similarities ranging from 94.35 % to 94.95 %, than to the Rotarix strain (92.34 %–93.09 %) (Table 2). Iranian P [4] strains, which belonged to lineage V, were rather distantly related to both Rotarix and RotaTeq P [6] strains compared to P [6] strains, with amino acid similarities of 85.61 %–85.92 % to the former and 87.27 %–87.41 % to the latter (Table 2). Additionally, the detected P [7] strains clustered in lineage I and displayed low amino acid resemblance, ranging from 70.07 % to 71.73 % when compared to P [6] strains of Rotarix and 68 %–69.61 % when compared to RotaTeq, respectively.

### 3.5 Comparative analysis of VP7 neutralizing epitopes between Iranian strains and vaccine strains

**Fig. 3:**
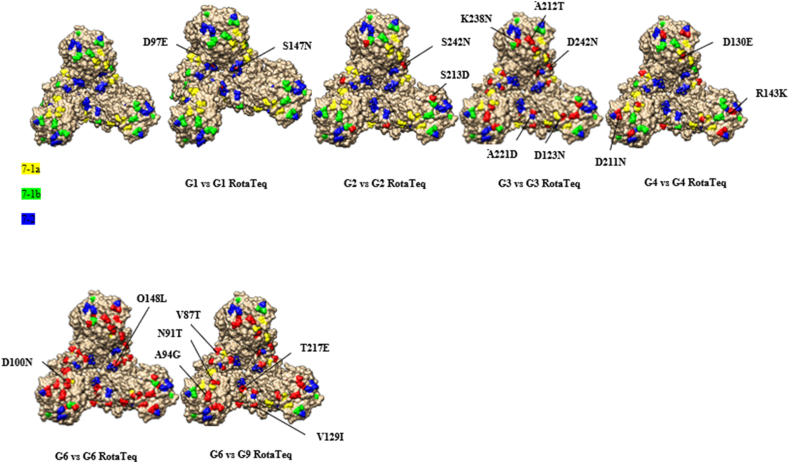
A three-dimensional illustration of the amino acid substitutions identified in the VP7 protein of RVA strains. VP7 3D structure (first image). Antigenic epitopes (7-1a, 7-1 b, and 7–2) are represented by yellow, green, and blue, respectively. Surface-exposed residues that differ between circulating strains in Iran and the strains contained in RotaTeq are shown in red.

The results of the amino acid comparison between the G-type strains in this study and the corresponding vaccine strains can be found in Table S1. When analyzing the VP7 protein, the isolates exhibited a relatively high percentage of amino acid variations across all subunits of the VP7 epitopes (Fig. 3). Among the 29 amino acid residues in the VP7 neutralizing epitopes as described by Zeller et al. [25], only five amino acids (positions 98, 104, 201, 264 and 291) were completely conserved among the circulating Iranian strains when compared to the vaccine strains. Upon aligning the Iranian G1 serotypes from this study with the G1 strains of RotaTeq and Rotarix vaccine strains, a substantial similarity in amino acid residues was observed. All the G1 strains displayed absolutely identical VP7 antigenic epitopes to Rotarix G1 strains, whereas two amino acid substitutions in 7-1a and 7–2 epitopes were found in all of them in comparison with G1 of RotaTeq (D97E and S147 N). Regarding the G2 strains, when compared to G2 of RotaTeq, there were few amino acid variations in antigenic epitopes in the 7-1a and 7-1 b epitopes (A87T, D96 N, S213D and S242 N). Conversely, this genotype showed 18 amino acid changes in all three antigenic regions when compared to G1 of Rotarix. In the case of G3, in comparison to RotaTeq, the G3 strains demonstrated three to five amino acid differences (see supplementary file 1). The OQ789845/G3/I strain showed the fewest substitutions (A212T, K238 N and D242 N) in the 7-1a and 7-1 b regions, while the OQ789859/G3/I strain displayed the most replacements (D123 N, A212T, K238 N, A221D and D242 N) across all regions. The remaining three strains had only four mutations (A212T, K238 N, A221D and D242 N) in the 7-1 b and 7–2 epitopes. Interestingly, each of these G3 strains contained the K238 N mutation, which provides a potential N-linked glycosylation site that does not exist at the corresponding position in RotaTeq G3 [25]. In addition, G3 showed 11–12 amino acid substitutions across all three epitopes when compared to G1 of Rotarix.

The VP7 epitopes of both G4 strains differed by four amino acid substitutions when compared to the epitopes in the G4 strain of RotaTeq (D130E, R143K, A145T, and D211 N), all of which were present in all three antigenic epitopes. Conversely, these strains had 16 amino acid discrepancies when compared to the G1 strain of Rotarix. As for the newly identified G6 strain, there were 7 and 15 amino acid distinctions compared to the G6 and G1 strains of RotaTeq and Rotarix in all three regions, respectively. Furthermore, Iranian G9 strains showed a total of 13 amino acid differences compared to RotaTeq G1-G4 and G6 strains, and a sum of 14 variations in comparison to G1 of Rotarix. These variances were found within the three neutralizing antigen epitopes (7-1a, 7-1 b, and 7–2).

### 3.6 Comparative analysis of VP8 neutralizing epitopes between Iranian strains and vaccine strains

**Fig. 4:**
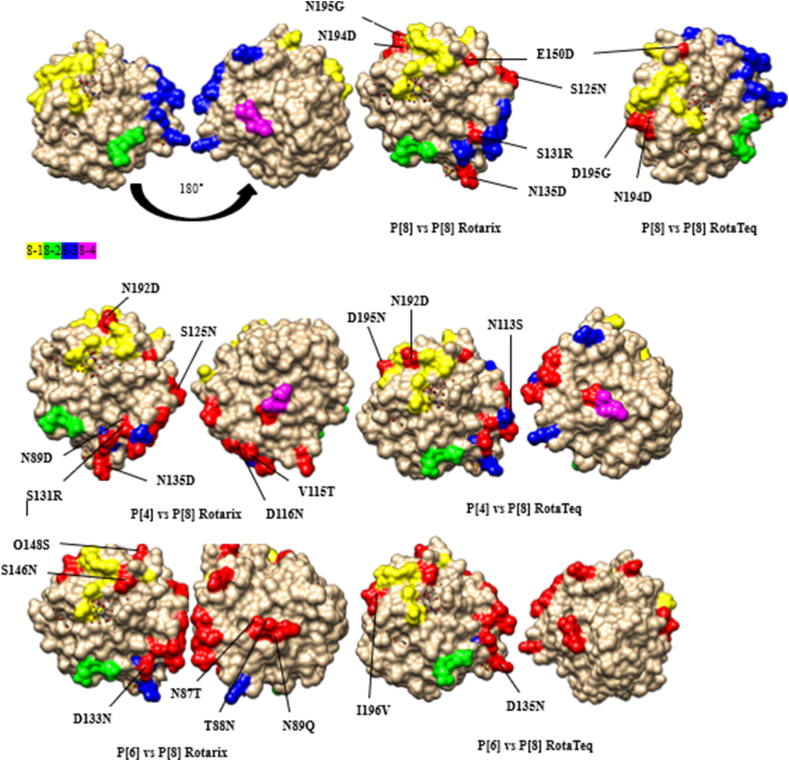
A three-dimensional illustration of the amino acid substitutions identified in the VP4 protein of RVA strains. VP4 3D structure (first image). Antigenic epitopes (8–1, 8–1, 8–3 and 8–4) are represented by yellow, green, blue, and purple respectively. Surface-exposed residues that differ between circulating strains in Iran and the strains contained in Rotarix or RotaTeq are shown in red.

VP4 typically cleaves into two fragments known as VP8 and VP5. The VP8 fragment encompasses four surface-exposed antigens, namely 8–1, 8–2, 8–3, and 8–4, which collectively consist of 25 amino acids [25]. All the Iranian P [6] strains shared 19 out of 25 residues that were identical to those present in the VP4 antigenic epitopes of Rotarix and RotaTeq. Notably, epitopes 8–2 and 8–4 were conserved among all of these genotypes (see supplementary file 2). It is important to note that all P [6] strains featured E150D and D195G/N195G substitutions, and four of them had an N194D mutation. This means that the polar asparagine in Rotarix and aspartic acid in RotaTeq have been replaced by a non-polar glycine. As for P [4] strains, there were a greater number of amino acid distinctions across three antigenic epitopes, namely 8–1, 8–3, and 8–4, compared to P [6] strains, with 10 and 11 amino acid discrepancies compared to RotaTeq and Rotarix P [6] strains, respectively. Furthermore, similar to P [6] strains, the 8–2 epitope was conserved among all P [4] strains. Upon mapping these variations within the VP4 epitopes of the vaccine strains and Iranian strains, it was revealed that they appeared relatively inconsistent along the molecule's front and back (Fig. 4). P [7] strains showed the most divergence from both P [6] vaccine strains, with 15 amino acid differences from Rotarix, and 16 from RotaTeq. These mutations were concentrated in the 8–3 and 8–4 epitopes.

### 3.7 Comparative analysis of cytotoxic T Lymphocyte epitopes of Iranian and vaccine strains

Among the 26 amino acids found in CTL epitopes, only five were completely conserved in Iranian G-types (21Y, 23 L, 24 K, 51Q, and 52 N) (see supplementary file 3). A comparison of G1 vaccine and Iranian strains revealed three amino acid differences in CTL epitopes at positions L19F, R28Q, and Y/T41S. The G2 serotypes differed by 4 and 14 amino acid substitutions from G1 of Rotarix and G2 of RotaTeq, respectively. Iranian G3 and G4 strains displayed the fewest number of substitutions within their CTL epitopes when compared to the G3 and G4 of RotaTeq, with only one mutation in the former and two discrepancies in the latter.

## 4 Discussion

Genetic drift, reassortment, animal-to-human transmission, and rearrangement are among mechanisms that lead to the emergence of novel rotavirus variants worldwide [26]. Members of known lineages in each genotype contain accumulated mutations that have been introduced in each replication cycle [25]. Some G and P genotypes exhibit significant amino acid differences in their antigenic epitopes compared to the vaccine strains, which might be responsible for escaping induced neutralizing antibodies [27]. In the present study, the amino acid sequences and antigenic epitopes of VP7 and VP4 of circulating Iranian strains were compared with Rotarix and RotaTeq vaccine strains to determine potential disparities that could affect vaccine efficacy and their implications for future vaccination programs.

Based on VP7 amino acid sequence analysis, G1 strains showed a high amino acid identity with G1 of RotaTeq (G1/II), but all of them displayed two amino acid changes in neutralizing epitopes, D97E and S147 N [28], which had been reported before in Serbian and Chinese G1 strains [21,29]. G2 strains are typically associated with P [4] genotypes, and protection against this genotype by RotaTeq primarily relies on the G2 (VP7) element of the vaccine. Some mutations in the neutralizing epitopes of this genotype are related to escape from vaccine-induced immunity. Accordingly, an amino acid alteration at position 96 in antigenic region 7-1a in G2P [4] led to an outbreak in Australia [30].

The study revealed the presence of a K238 N amino acid substitution in all the G3 strains, which may indicate an N-linked glycosylation site, consistent with results reported in China, Qatar and Italy [20,21,31]. The K238 N mutation can enhance viral replication, diminish the efficacy of monoclonal antibodies, and prevent neutralizing antibody activity [32,33]. Furthermore, the shift in charge may impact the chemical properties of the protein, and the change in polarity implies that the epitope may become more inaccessible due to its increased hydrophobicity [34]. Epidemiological data from Australia obtained during the post-vaccine era suggest a possible increase in G3 strains where RotaTeq has been administered [35]. In addition, after the introduction of RotaTeq in the United States, the G3 genotype became predominant in some seasons [36].

Four discrepancies were found between the RotaTeq and Iranian G4 antigenic epitopes. The existence of these mutations among Iranian RVA-G4 strains may indicate the emergence of a variant with the capacity for immune escape [19]. Additionally, an alignment of their VP7 amino acids revealed the insertion of an asparagine residue at position 76, which can affect glycosylation and, in turn, modify antigenic characteristics. This is because the asparagine insertion at this position, situated in a hydrophilic region, has the potential to enhance the region's hydrophilicity [37].

Regarding the G6 and G9 Iranian strains, both were distantly related to Rotarix and RotaTeq VP7 strains, with G9 strains showing a relatively closer similarity to G3 of RotaTeq. This was consistent with a previous study in Iran [19]. A comparison of the VP7 epitopes of the G6 strain to the G6 of RotaTeq showed seven amino acid differences distributed across all three antigenic epitopes, which did not represent any radical changes in the antigenicity of VP7 epitopes, as previously reported in a US study [33]. As for the five G9 strains in this study, there was a high frequency of mutations across all three regions when compared to VP7 regions of both vaccines. One G9 strain (OQ789847/G9/III) exhibited a D100 N mutation, which, as reported in Chinese G9 strains, represents an escape mutant [38].A comparison of combined genotypes for the strains representing the G9 segment showed fully heterotypic genotypes in four (G9P [4] = 1, G9P [7] = 3), and partially heterotypic in one of the sequenced strains (G9P [6] = 1). Although, in our study, sequencing of both G9 and P [7] for RVA strains was done for just one strain (OQ789885), its comparative analysis showed the highest discrepancy in amino acid sequences compared to RotaTeq and Rotarix vaccines (12 and 13 amino acid substitutions for G9, and 16 and 15 substitutions for P [7], respectively). The co-existing difference in neutralizing epitopes of VP4 and VP7 in genotypes with over 50 % diversity, as was shown in G9P [7] strain, highlights the risk of immune escape after the implementation of vaccination program with RotaTeq and Rotarix. Notably, this genotype is the least similar to the vaccine genotypes, which we speculate is the reason for its emergence in countries that have implemented RVA vaccination within their vaccine programs [39].

The VP4 spike protein plays a crucial role in viral neutralization due to its several structural and functional roles, including virus particle binding, penetration, and maturation [29]. Similar patterns of amino acid substitutions in VP8* epitopes of the P [6] strains between circulating and vaccine strains in our study were reported from Serbia, China and Qatar [20,21,29]. The amino acid changes identified at positions S131R and N135D in this study could result in polarity changes, which play a role in RV's escape from the host immunity [29]. Taken together, although both vaccines have shown to be very effective in Europe and the United States, there is increasing concern about the evolution of resistant strains [40]. Regarding the high diversity of RVA genotypes in Iran and detected mutations in most of their neutralizing epitopes, escape from the induced immunity of RotaTeq and Rotarix seems to be highly probable after the vaccination program. However, as was shown in a study in Belgium, the introduction of the vaccine can effectively decrease dominant genotypes from the same lineage, G1/II-P [6]/I strains, compared to unrelated lineages [25], which could highly reduce the infection rate among children.

Presentation of a specific amino acid sequence of structural antigens to B- or T-cells can mediate immunity to RVA. Although the precise mechanism by which vaccination confers protection against rotavirus is not well known, neutralizing antibodies specific to VP4 and VP7 proteins seem to be the key factors of protection, which are considered for the development of the RotaTeq vaccines [40]. In this study, the similarity of the two known CTL epitopes of the VP7 protein was analyzed in the Iranian G-types compared with Rotarix and RotaTeq strains. G1 strains in our isolates showed 3 amino acid substitutions within VP7 CTL epitopes compared to the vaccine strains, which is consistent with a previous study in Africa [41]. In the case of other genotypes, in comparison to the RotaTeq strain, G2 strains showed the highest number of discrepancies, while G3 and G4 strains displayed the least number of differences across both epitopes. This discrepancy was also reported in a study in Russia [42]. According to our knowledge, there are no available data on the effects of these mutations on the immunity induced by CTLs. The impact of the characterized mutations in VP7 CTL epitopes of the circulating strains in Iran on the processing and presentation of RVA antigens to immune cells and RVA clearance should be further studied.

This study had some limitations. The isolates analyzed in this study belonged to pediatric patients admitted to Mofid Children's Hospital in Tehran. The sample size was relatively small, which may not allow a deep insight into the RVA lineages circulating in Iran. To obtain a better overview of lineage diversity for effective vaccine implementation, a comprehensive multicenter study is necessary. Additionally, while the data were sufficient for differentiating RVA genotypes and their lineages, sub-genotypic lineages could not be identified. This was primarily due to the partial sequencing of VP7 and VP4 gene segments and budgetary constraints.

## 5 Conclusion

This study provides crucial insights into the genetic and antigenic characteristics of Iranian strains compared with the vaccine strains. Given the high diversity of RVA genotypes in Iran and detected mutations in neutralizing and CTL epitopes, we speculate that escape from the induced immunity of RotaTeq and Rotarix might be probable after vaccination. To fully demonstrate the importance of these differences and their implications on vaccine efficacy, further studies on the intragenotype antigenic variability of RVA are necessary before the introduction of Rotarix and RotaTeq into the national immunization program.

## Funding details

This work was supported by the 10.13039/100004423World Health Organization under grant 202700981.

## Ethics approval and consent to participate

This study was approved by the research ethics committees of Research Institute of Children's Health at Shahid Beheshti University of Medical Sciences (IR.SBMU.RICH.REC.1401.021), and parental consent was obtained for all admitted children.

## Data availability

The sequencing files have been deposited in the NCBI database under the following accession numbers: OQ789844-OQ789866 and OQ789867- OQ789894.

## CRediT authorship contribution statement

**Tina Fallah:** Writing – original draft, Formal analysis. **Roxana Mansour Ghanaiee:** Supervision, Methodology, Investigation, Conceptualization. **Abdollah Karimi:** Supervision, Methodology, Investigation, Conceptualization. **Seyed Mohsen Zahraei:** Funding acquisition. **Sussan Mahmoudi:** Funding acquisition. **Masoud Alebouyeh:** Writing – review & editing, Supervision, Methodology, Investigation, Conceptualization.

## Declaration of competing interest

The authors declare that they have no known competing financial interests or personal relationships that could have appeared to influence the work reported in this paper.
