# Supplementary material for: Comparative analysis of the RVA VP7 and VP4 antigenic epitopes circulating in Iran and the Rotarix and RotaTeq vaccines
Source: Heliyon. 2024 Jul 4;10(13):e33887. doi: 10.1016/j.heliyon.2024.e33887 (PMC11282978; doi:10.1016/j.heliyon.2024.e33887)
Supplement: Multimedia component 1 [file mmc1.docx]

**Table S1.** Alignment of Antigenic Residues in VP7 among Strains in Rotarix, RotaTeq, and Circulating Iranian Strains.^a^

| Rotavirus genotype/lineage | Neutralizing epitopes of the VP7 | | | | | | | | | | | | | | | | | | | | | | | | | | | | | | | | | | | | | | | | | | | | | | | | |
| --- | --- | --- | --- | --- | --- | --- | --- | --- | --- | --- | --- | --- | --- | --- | --- | --- | --- | --- | --- | --- | --- | --- | --- | --- | --- | --- | --- | --- | --- | --- | --- | --- | --- | --- | --- | --- | --- | --- | --- | --- | --- | --- | --- | --- | --- | --- | --- | --- | --- |
|  | 7-1a | | | | | | | | | | | | | | | | | | | | | | 7-1b | | | | | | | | | 7-2 | | | | | | | | | | | | | | | | | |
|  | 87 | | | 91 | 94 | | 96 | 97 | | 98 | 99 | | 100 | 104 | | 123 | 125 | | 129 | 130 | | 291 | 201 | 211 | | 212 | | 213 | 238 | | 242 | | | 143 | 145 | | 146 | 147 | | | 148 | 190 | | | 217 | 221 | | | 264 |
| Rotarix G1/II | T | | T | | N | G | | E | W | | K | D | | Q | S | | V | V | | D | K | | Q | | N | V | D | | N | T | | K | | | D | Q | | N | | L | | S | | M | | N | | G | |
| RotaTeq G1/III | T | T | | | N | G | | D | W | | K | D | | Q | S | | V | V | | D | K | | Q | | N | V | D | | N | T | | K | | | D | Q | | S | L | | | S | M | | | | N | | G |
| [OQ789865](https://www.ncbi.nlm.nih.gov/nuccore/OQ789865.1)/G1/II | * | * | | | * | * | | E | * | | * | * | | * | * | | * | * | | * | * | | * | | * | * | * | | * | * | | * | | | * | * | | N | * | | | * | * | | | | * | | * |
| [OQ789861](https://www.ncbi.nlm.nih.gov/nuccore/OQ789865.1)/G1/II | * | * | | | * | * | | E | * | | * | * | | * | * | | * | * | | * | * | | * | | * | * | * | | * | * | | * | | | * | * | | N | * | | | * | * | | | | * | | * |
| [OQ789860](https://www.ncbi.nlm.nih.gov/nuccore/OQ789865.1)/G1/II | * | * | | | * | * | | E | * | | * | * | | * | * | | * | * | | * | * | | * | | * | * | * | | * | * | | * | | | * | * | | N | * | | | * | * | | | | * | | * |
| [OQ789854](https://www.ncbi.nlm.nih.gov/nuccore/OQ789865.1)/G1/II | * | * | | | * | * | | E | * | | * | * | | * | * | | * | * | | * | * | | * | | * | * | * | | * | * | | * | | | * | * | | N | * | | | * | * | | | | * | | * |
| [OQ789853](https://www.ncbi.nlm.nih.gov/nuccore/OQ789865.1)/G1/II | * | * | | | * | * | | E | * | | * | * | | * | * | | * | * | | * | * | | * | | * | * | * | | * | * | | * | | | * | * | | N | * | | | * | * | | | | * | | * |
| [OQ789851](https://www.ncbi.nlm.nih.gov/nuccore/OQ789865.1)/G1/II | * | * | | | * | * | | E | * | | * | * | | * | * | | * | * | | * | * | | * | | * | * | * | | * | * | | * | | | * | * | | N | * | | | * | * | | | | * | | * |
| [OQ789850](https://www.ncbi.nlm.nih.gov/nuccore/OQ789865.1)/G1/II | * | * | | | * | * | | E | * | | * | * | | * | * | | * | * | | * | * | | * | | * | * | * | | * | * | | * | | | * | * | | N | * | | | * | * | | | | * | | * |
| [OQ789846](https://www.ncbi.nlm.nih.gov/nuccore/OQ789865.1)/G1/II | * | * | | | * | * | | E | * | | * | * | | * | * | | * | * | | * | * | | * | | * | * | * | | * | * | | * | | | * | * | | N | * | | | * | * | | | | * | | * |
| [OQ789844](https://www.ncbi.nlm.nih.gov/nuccore/OQ789865.1)/G1/II | * | * | | | * | * | | E | * | | * | * | | * | * | | * | * | | * | * | | * | | * | * | * | | * | * | | * | | | * | * | | N | * | | | * | * | | | | * | | * |
|  | | | | | | | | | | | | | | | | | | | | | | |  | | | | | | | | |  | | | | | | | | | | | | | | | | | |
| RotaTeq G2/II | A | N | | | S | D | | E | W | | E | N | | Q | D | | T | M | | N | K | | Q | | D | V | S | | N | S | | R | | | D | N | | T | S | | | D | I | | | | S | | G |
| [OQ789862](https://www.ncbi.nlm.nih.gov/nuccore/OQ789865.1)/G2/IV | T | N | | | S | N | | * | * | | E | N | | * | D | | T | M | | N | * | | * | | D | * | D | | * | N | | R | | | * | N | | T | S | | | D | I | | | | S | | * |
|  | | | | | | | | | | | | | | | | | | | | | | |  | | | | | | | | | |  | | | | | | | | | | | | | | | | |
| RotaTeq G3/II | T | T | | | N | N | | S | W | | K | D | | Q | D | | A | V | | D | K | | Q | | D | A | N | | K | D | | K | | | D | A | | T | L | | | S | E | | | | A | | G |
| [OQ789859](https://www.ncbi.nlm.nih.gov/nuccore/OQ789865.1)/G3/I | * | * | | | * | N | | S | * | | * | * | | * | N | | A | * | | * | * | | * | | D | T | N | | N | N | | * | | | * | A | | T | * | | | * | E | | | | D | | * |
| [OQ789857](https://www.ncbi.nlm.nih.gov/nuccore/OQ789865.1)/G3/I | * | * | | | * | N | | S | * | | * | * | | * | D | | A | * | | * | * | | * | | D | T | N | | N | N | | * | | | * | A | | T | * | | | * | E | | | | D | | * |
| [OQ789852](https://www.ncbi.nlm.nih.gov/nuccore/OQ789865.1)/G3/I | * | * | | | * | N | | S | * | | * | * | | * | D | | A | * | | * | * | | * | | D | T | N | | N | N | | * | | | * | A | | T | * | | | * | E | | | | D | | * |
| [OQ789845](https://www.ncbi.nlm.nih.gov/nuccore/OQ789865.1)/G3/I | * | * | | | * | N | | S | * | | * | * | | * | D | | A | * | | * | * | | * | | D | T | N | | N | N | | * | | | * | A | | T | * | | | * | E | | | | A | | * |
| [OQ789848](https://www.ncbi.nlm.nih.gov/nuccore/OQ789865.1)/G3/I | * | * | | | * | N | | S | * | | * | * | | * | D | | A | * | | * | * | | * | | D | T | N | | N | N | | * | | | * | A | | T | * | | | * | E | | | | D | | * |
|  | | | | | | | | | | | | | | | | | | | | | | |  | | | | | | | | |  | | | | | | | | | | | | | | | | | |
| RotaTeq G4/I | S | T | | | S | T | | E | W | | K | D | | Q | N | | L | I | | D | K | | Q | | D | T | A | | D | T | | R | | | A | S | | G | E | | | S | T | | | | S | | G |
| [OQ789849](https://www.ncbi.nlm.nih.gov/nuccore/OQ789865.1)/G4/I | S | * | | | S | T | | * | * | | * | * | | * | N | | L | I | | E | * | | * | | N | T | A | | D | * | | K | | | T | S | | G | E | | | * | T | | | | S | | * |
| [OQ789858](https://www.ncbi.nlm.nih.gov/nuccore/OQ789865.1)/G4/I | S | * | | | S | T | | * | * | | * | * | | * | N | | L | I | | E | * | | * | | N | T | A | | D | * | | K | | | T | S | | G | E | | | * | T | | | | S | | * |
|  | | | | | | | | | | | | | | | | | | | | | | |  | | | | | | | | |  | | | | | | | | | | | | | | | | | |
| RotaTeq G6 | V | N | | | A | T | | E | W | | K | D | | Q | D | | A | V | | E | K | | Q | | N | P | D | | N | A | | K | | | D | S | | T | Q | | | S | T | | | | T | | G |
| [OQ789862](https://www.ncbi.nlm.nih.gov/nuccore/OQ789865.1)/G6 | V | N | | | A | T | | * | * | | * | N | | * | D | | A | * | | D | * | | * | | D | P | N | | D | T | | * | | | * | S | | T | L | | | * | T | | | | T | | * |
| [OQ789866](https://www.ncbi.nlm.nih.gov/nuccore/OQ789865.1)/G9/III | T | T | | | G | T | | * | * | | * | * | | * | D | | A | I | | D | * | | * | | * | T | A | | D | N | | * | | | * | S | | T | L | | | * | E | | | | S | | * |
| [OQ789856](https://www.ncbi.nlm.nih.gov/nuccore/OQ789865.1)/G9/III | T | T | | | G | T | | * | * | | * | * | | * | D | | A | I | | D | * | | * | | * | T | A | | D | N | | * | | | * | S | | T | L | | | * | E | | | | S | | * |
| [OQ789864](https://www.ncbi.nlm.nih.gov/nuccore/OQ789865.1)/G9/III | T | T | | | G | T | | * | * | | * | * | | * | D | | A | I | | D | * | | * | | * | T | A | | D | N | | * | | | * | S | | T | L | | | * | E | | | | S | | * |
| [OQ789863](https://www.ncbi.nlm.nih.gov/nuccore/OQ789865.1)/G9/III | T | T | | | G | T | | * | * | | * | * | | * | D | | A | I | | D | * | | * | | * | T | A | | D | N | | * | | | * | S | | T | L | | | * | E | | | | S | | * |
| [OQ789847](https://www.ncbi.nlm.nih.gov/nuccore/OQ789865.1)/G9/III | T | T | | | G | T | | * | * | | * | N | | * | D | | A | I | | D | * | | * | | * | T | A | | D | N | | * | | | * | S | | T | L | | | * | E | | | | S | | * |

1. Antigenic Residues are categorized into three epitopes (7-1a, 7-1b, and 7-2). Amino acids that differ from Rotarix are highlighted in blue, residues that differ from RotaTeq are highlighted in green, and those that differ from both vaccines are marked in red. [*], Same as Rotarix and RotaTeq.
