# Supplementary material for: Comparative analysis of the RVA VP7 and VP4 antigenic epitopes circulating in Iran and the Rotarix and RotaTeq vaccines
Source: Heliyon. 2024 Jul 4;10(13):e33887. doi: 10.1016/j.heliyon.2024.e33887 (PMC11282978; doi:10.1016/j.heliyon.2024.e33887)
Supplement: Multimedia component 2 [file mmc2.docx]

**Table S2.** Alignment of Antigenic Residues in VP4 among Strains in Rotarix, RotaTeq, and Circulating Iranian Strains.^a^

| Rotavirus genotype/lineage | Neutralizing epitopes of the VP4 | | | | | | | | | | | | | | | | | | | | | | | | | | | | | | | | | |
| --- | --- | --- | --- | --- | --- | --- | --- | --- | --- | --- | --- | --- | --- | --- | --- | --- | --- | --- | --- | --- | --- | --- | --- | --- | --- | --- | --- | --- | --- | --- | --- | --- | --- | --- |
|  | 8-1 | | | | | | | | | | | | | | | | | | 8-2 | | 8-3 | | | | | | | | | 8-4 | | | | |
|  | 100 | 146 | | 148 | | 150 | | 188 | | 190 | | | 192 | 193 | | 194 | 195 | 196 | 180 | 183 | 113 | 114 | 115 | 116 | 125 | 131 | 132 | 133 | 135 | 87 | 88 | | 89 | |
| Rotarix P[8]/I | D | | S | | Q | | E | | S | | T | N | | | L | N | N | I | T | A | N | P | V | D | S | S | N | D | N | N | | T | | N |
| RotaTeq P[8]/II | D | | S | | Q | | E | | S | | T | N | | | L | N | D | I | T | A | N | P | V | D | N | R | N | D | D | N | | T | | N |
| OQ789894/P[8]/III | * | | * | | * | | D | | * | | * | * | | | * | D | G | * | * | * | * | * | * | * | N | R | * | * | D | * | | * | | * |
| OQ789892/P[8]/III | * | | * | | * | | D | | * | | * | * | | | * | * | G | * | * | * | * | * | * | * | N | R | * | * | D | * | | * | | * |
| OQ789891/P[8]/III | * | | * | | * | | D | | * | | * | * | | | * | * | G | * | * | * | * | * | * | * | N | R | * | * | D | * | | * | | * |
| OQ789889/P[8]/III | * | | * | | * | | D | | * | | * | * | | | * | D | G | * | * | * | * | * | * | * | N | R | * | * | D | * | | * | | * |
| OQ789882/P[8]/III | * | | * | | * | | D | | * | | * | * | | | * | * | G | * | * | * | * | * | * | * | N | R | * | * | D | * | | * | | * |
| OQ789881/P[8]/III | * | | * | | * | | D | | * | | * | * | | | * | * | G | * | * | * | * | * | * | * | N | R | * | * | D | * | | * | | * |
| OQ789880/P[8]/III | * | | * | | * | | D | | * | | * | * | | | * | * | G | * | * | * | * | * | * | * | N | R | * | * | D | * | | * | | * |
| OQ789875/P[8]/III | * | | * | | * | | D | | * | | * | * | | | * | D | G | * | * | * | * | * | * | * | N | R | * | * | D | * | | * | | * |
| OQ789874/P[8]/III | * | | * | | * | | D | | * | | * | * | | | * | D | G | * | * | * | * | * | * | * | N | R | * | * | D | * | | * | | * |
| OQ789873/P[8]/III | * | | * | | * | | D | | * | | * | * | | | * | * | G | * | * | * | * | * | * | * | N | R | * | * | D | * | | * | | * |
| OQ789872/P[8]/III | * | | * | | * | | D | | * | | * | * | | | * | * | G | * | * | * | * | * | * | * | N | R | * | * | D | * | | * | | * |
| OQ789866/P[8]/III | * | | * | | * | | D | | * | | * | * | | | * | * | G | * | * | * | * | * | * | * | N | R | * | * | D | * | | * | | * |
| OQ789869/P[8]/III | * | | * | | * | | D | | * | | * | * | | | * | * | G | * | * | * | * | * | * | * | N | R | * | * | D | * | | * | | * |
| OQ789890/P[4]/IV | * | | * | | * | | D | | * | | * | D | | | * | * | N | * | * | * | S | * | T | N | N | E | * | S | D | * | | * | | D |
| OQ789887/P[4]/IV | * | | * | | * | | D | | * | | * | D | | | * | * | N | * | * | * | S | * | T | N | N | E | * | S | D | * | | * | | D |
| OQ789883/P[4]/IV | * | | * | | * | | D | | * | | * | D | | | * | * | N | * | * | * | S | Q | T | N | N | E | * | S | D | * | | * | | D |
| OQ789879/P[4]/IV | * | | * | | * | | D | | * | | * | D | | | * | * | N | * | * | * | S | * | T | N | N | E | * | S | D | * | | * | | D |
| OQ789877/P[4]/IV | * | | * | | * | | D | | * | | * | D | | | * | * | N | * | * | * | S | * | T | N | N | E | * | S | D | * | | * | | D |
| OQ789893/P[6]/I | * | | N | | S | | * | | * | | * | * | | | * | S | E | V | * | * | T | N | Q | S | V | E | * | N | N | T | | N | | Q |
| OQ789888/P[6]/I | * | | N | | S | | * | | * | | * | * | | | * | S | E | V | * | * | T | N | Q | S | V | E | * | N | N | T | | N | | Q |
| OQ789886/P[6]/I | * | | N | | S | | * | | * | | * | * | | | * | S | E | V | * | * | T | N | Q | S | V | E | * | N | N | T | | N | | Q |
| OQ789885/P[6]/I | * | | N | | S | | * | | * | | * | * | | | * | S | E | V | * | * | T | N | Q | S | V | E | * | N | N | T | | N | | Q |
| OQ789884/P[6]/I | * | | N | | S | | * | | * | | * | * | | | * | S | E | V | * | * | T | N | Q | S | V | E | * | N | N | T | | N | | Q |
| OQ789878/P[6]/I | * | | * | | N | | * | | * | | * | * | | | * | S | E | V | * | * | T | N | Q | N | T | E | * | N | N | T | | N | | Q |
| OQ789876/P[6]/I | * | | N | | S | | * | | * | | * | * | | | * | S | E | V | * | * | T | N | Q | S | V | E | * | N | N | T | | N | | Q |
| OQ789871/P[6]/I | * | | N | | S | | * | | * | | * | * | | | * | S | E | V | * | * | T | N | Q | S | V | E | * | N | N | T | | N | | Q |
| OQ789870/P[6]/I | * | | N | | S | | * | | * | | * | * | | | * | S | E | V | * | * | T | N | Q | S | V | E | * | N | N | T | | N | | Q |
| OQ789868/P[6]/I | * | | N | | S | | * | | * | | * | * | | | * | S | E | V | * | * | T | N | Q | S | V | E | * | N | N | T | | N | | Q |

1. Antigenic Residues are categorized into four epitopes (8-1, 8-2, 8-3 and 8-4). Amino acids that differ from Rotarix are highlighted in blue, residues that differ from RotaTeq are highlighted in green, and those that differ from both vaccines are marked in red. [*], Same as Rotarix and RotaTeq.
