# Supplementary material for: Comparative analysis of the RVA VP7 and VP4 antigenic epitopes circulating in Iran and the Rotarix and RotaTeq vaccines
Source: Heliyon. 2024 Jul 4;10(13):e33887. doi: 10.1016/j.heliyon.2024.e33887 (PMC11282978; doi:10.1016/j.heliyon.2024.e33887)
Supplement: Multimedia component 3 [file mmc3.docx]

**Table S3.** Alignment of Antigenic Residues in T-Cell Antigen Epitopes of Circulating Iranian VP7 Strains and Rotarix and RotaTeq Vaccine Strains.^a^

| Rotavirus genotype/lineage | T-cell epitopes | | | | | | | | | | | | | | | | | | | | | | |  | | | | | | | | | | | | | | | | | | | | | | | | | |
| --- | --- | --- | --- | --- | --- | --- | --- | --- | --- | --- | --- | --- | --- | --- | --- | --- | --- | --- | --- | --- | --- | --- | --- | --- | --- | --- | --- | --- | --- | --- | --- | --- | --- | --- | --- | --- | --- | --- | --- | --- | --- | --- | --- | --- | --- | --- | --- | --- | --- |
|  | T-cell epitope 16-28 aa | | | | | | | | | | | | | | | | | | | | | | | T-cell epitope 40-52 aa | | | | | | | | | | | | | | | | | | | | | | | | | |
|  | 16 | | | 17 | 18 | | 19 | 20 | | 21 | 22 | | 23 | 24 | | 25 | 26 | | 27 | 28 | | | 40 | | 41 | | 42 | | 43 | | 44 | | 45 | | 46 | | 47 | | 48 | | 49 | | | 50 | | 51 | | | 52 |
| Rotarix G1/II | I | | I | | L | L | | N | Y | | I | L | | K | S | | V | T | | R | | I | | | Y | V | | A | | L | | F | | A | | L | | T | | R | | | A | | Q | | | N | |
| RotaTeq G1/III | I | I | | | L | L | | N | Y | | I | L | | K | S | | V | T | | R | | I | | | T | V | | A | | L | | F | | A | | L | | T | | R | | A | | | Q | | N | | |
| [OQ789865](https://www.ncbi.nlm.nih.gov/nuccore/OQ789865.1)/G1/II | * | * | | | * | F | | * | * | | * | * | | * | * | | * | * | | Q | | * | | | S | * | | * | | * | | * | | * | | * | | * | | * | | * | | | * | | * | | |
| [OQ789861](https://www.ncbi.nlm.nih.gov/nuccore/OQ789865.1)/G1/II | * | * | | | * | F | | * | * | | * | * | | * | * | | * | * | | Q | | * | | | S | * | | * | | * | | * | | * | | * | | * | | * | | * | | | * | | * | | |
| [OQ789860](https://www.ncbi.nlm.nih.gov/nuccore/OQ789865.1)/G1/II | * | * | | | * | F | | * | * | | * | * | | * | * | | * | * | | Q | | * | | | S | * | | * | | * | | * | | * | | * | | * | | * | | * | | | * | | * | | |
| [OQ789854](https://www.ncbi.nlm.nih.gov/nuccore/OQ789865.1)/G1/II | * | * | | | * | F | | * | * | | * | * | | * | * | | * | * | | Q | | * | | | S | * | | * | | * | | * | | * | | * | | * | | * | | * | | | * | | * | | |
| [OQ789853](https://www.ncbi.nlm.nih.gov/nuccore/OQ789865.1)/G1/II | * | * | | | * | F | | * | * | | * | * | | * | * | | * | * | | Q | | * | | | S | * | | * | | * | | * | | * | | * | | * | | * | | * | | | * | | * | | |
| [OQ789851](https://www.ncbi.nlm.nih.gov/nuccore/OQ789865.1)/G1/II | * | * | | | * | F | | * | * | | * | * | | * | * | | * | * | | Q | | * | | | S | * | | * | | * | | * | | * | | * | | * | | * | | * | | | * | | * | | |
| [OQ789850](https://www.ncbi.nlm.nih.gov/nuccore/OQ789865.1)/G1/II | * | * | | | * | F | | * | * | | * | * | | * | * | | * | * | | Q | | * | | | S | * | | * | | * | | * | | * | | * | | * | | * | | * | | | * | | * | | |
| [OQ789846](https://www.ncbi.nlm.nih.gov/nuccore/OQ789865.1)/G1/II | * | * | | | * | F | | * | * | | * | * | | * | * | | * | * | | Q | | * | | | S | * | | * | | * | | * | | * | | * | | * | | * | | * | | | * | | * | | |
| [OQ789844](https://www.ncbi.nlm.nih.gov/nuccore/OQ789865.1)/G1/II | * | * | | | * | F | | * | * | | * | * | | * | * | | * | * | | Q | | * | | | S | * | | * | | * | | * | | * | | * | | * | | * | | * | | | * | | * | | |
|  | | | | | | | | | | | | | | | | | | | | |  | | | | | | | | | | | | | | | | | | | | | | | | | | | | |
| RotaTeq G2/II | I | I | | | L | L | | N | Y | | I | L | | K | T | | I | T | | N | | V | | | I | V | | L | | I | | S | | P | | F | | V | | R | | T | | | Q | | N | | |
| [OQ789862](https://www.ncbi.nlm.nih.gov/nuccore/OQ789865.1)/G2/IV | * | T | | | * | * | | * | * | | * | * | | * | T | | I | * | | N | | L | | | I | A | | L | | M | | S | | P | | F | | V | | * | | T | | | * | | * | | |
|  | | | | | | | | | | | | | | | | | | | | |  | | | | | | | | | | | | | | | | | | | | | | | | | | | | |
| RotaTeq G3/II | V | I | | | L | L | | N | Y | | V | L | | K | S | | L | T | | R | | I | | | I | V | | I | | L | | S | | P | | T | | L | | N | | A | | | Q | | N | | |
| [OQ789859](https://www.ncbi.nlm.nih.gov/nuccore/OQ789865.1)/G3/I | V | * | | | * | * | | * | * | | V | * | | * | * | | L | * | | * | | * | | | I | * | | I | | * | | S | | P | | L | | L | | N | | * | | | * | | * | | |
| [OQ789857](https://www.ncbi.nlm.nih.gov/nuccore/OQ789865.1)/G3/I | V | * | | | * | * | | * | * | | V | * | | * | * | | L | * | | * | | * | | | I | * | | I | | * | | S | | P | | L | | L | | N | | * | | | * | | * | | |
| [OQ789852](https://www.ncbi.nlm.nih.gov/nuccore/OQ789865.1)/G3/I | V | * | | | * | * | | * | * | | V | * | | * | * | | L | * | | * | | * | | | I | * | | I | | * | | S | | P | | L | | L | | N | | * | | | * | | * | | |
| [OQ789845](https://www.ncbi.nlm.nih.gov/nuccore/OQ789865.1)/G3/I | V | * | | | * | * | | * | * | | V | * | | * | * | | L | * | | * | | * | | | I | * | | I | | * | | S | | P | | L | | L | | N | | * | | | * | | * | | |
| [OQ789848](https://www.ncbi.nlm.nih.gov/nuccore/OQ789865.1)/G3/I | V | * | | | * | * | | * | * | | V | * | | * | * | | L | * | | * | | * | | | I | * | | I | | * | | S | | P | | L | | L | | N | | * | | | * | | * | | |
|  | | | | | | | | | | | | | | | | | | | | |  | | | | | | | | | | | | | | | | | | | | | | | | | | | | |
| RotaTeq G4/I | F | V | | | L | V | | S | Y | | I | L | | K | T | | M | I | | K | | V | | | I | V | | V | | L | | S | | V | | L | | S | | N | | A | | | Q | | N | | |
| [OQ789849](https://www.ncbi.nlm.nih.gov/nuccore/OQ789865.1)/G4/I | F | V | | | F | * | | S | * | | * | * | | * | T | | I | I | | K | | V | | | I | * | | V | | * | | S | | V | | * | | S | | N | | * | | | * | | * | | |
| [OQ789858](https://www.ncbi.nlm.nih.gov/nuccore/OQ789865.1)/G4/I | F | V | | | F | * | | S | * | | * | * | | * | T | | I | I | | K | | V | | | I | * | | V | | * | | S | | V | | * | | S | | N | | * | | | * | | * | | |

1. Residues that differ from Rotarix are indicated in blue, those different from RotaTeq are highlighted in green, and those differing from both are marked in red. [*], Same as Rotarix and RotaTeq.
